# Vertical Alignment of Liquid Crystals Over a Functionalized Flexible Substrate

**DOI:** 10.1038/s41598-018-27039-3

**Published:** 2018-06-11

**Authors:** B. Sivaranjini, R. Mangaiyarkarasi, V. Ganesh, S. Umadevi

**Affiliations:** 1 Department of Industrial Chemistry, Alagappa University, Karaikudi, 630003 India; 2 Electrodics and Electrocatalysis Division (EEC), CSIR – Central Electrochemical Research Institute (CSIR−CECRI), Karaikudi, 630003 India

## Abstract

A simple and effective approach for vertical alignment of liquid crystals (LCs) over a functionalized transparent flexible substrate is described. Surface characterization of this commercially available plastic substrate through X-ray photoelectron spectroscopy (XPS) and attenuated total reflection infrared spectroscopy (ATR-IR) indicated that cellulose acetate is main component of the transparent substrate. This substrate was chemically functionalized with a suitable LC compound. A trimethoxysilane terminated new rod-shaped mesogen is synthesized and covalently attached to the pre-treated film through silane condensation reaction. LC functionalization of the polymer film is confirmed through contact angle (CA), atomic force microscopy (AFM), XPS and ATR-IR spectroscopy studies. Versatility of the LC modified flexible substrates for the alignment of bulk LC sample at substrate-LC interface was assessed for nematic (N) and smectic A (SmA) phases. Remarkably, LC functionalized cellulose acetate films were found to be highly efficient in assisting a perfect homeotropic alignment of LCs (for both, a room temperature N and a high temperature SmA phase) over the entire area of the LC sample under observation indicating their superior aligning ability in comparison to their unmodified and octadecyltrimethoxysilane (OTS) modified counterparts. The demonstrated method of surface modification of flexible polymer film is easy, surface modified substrates are stable for several months, retained their aligning ability intact and more importantly they are reusable with maximum delivery.

## Introduction

Liquid crystals (LCs) are self-assembled soft materials with interesting applications in a variety of fields such as display industry, sensing, biomedical, photonics, optoelectronics, electronic conductors, photovoltaics etc^1–8^. A pre-oriented well aligned sample of LC over a suitable substrate is highly crucial for most of these applications. For example, homeotropic alignment wherein long axis of the LC molecules (director) are oriented perpendicular to the substrate surface^9^, or planar alignment where LC director lie parallel the substrate surface^10^. In general, a vertical alignment of LC at a solid-LC interface is often preferred for sensor applications. Molecular orientation in LCs is very sensitive to small perturbations and any binding events which change the orientation of the molecules will be propagated through several micrometers to provide detectable optical signals in the form of change in the textures, which can be observed under a polarizing optical microscopy (POM). Thus a pre-oriented uniform film of LCs supported on a substrate can be effectively utilized for LC based sensing which is simple, rapid and an inexpensive process. Upto date, LC-based sensors for gas-phase analytes (such as organophosphonates, organoamines, aldehydes and organosulphur compounds) biomolecules (such as proteins, oligopeptides and DNA) and microorganisms (viruses, bacteria and mammalian cells) have been described^4,5^.

The conventional method followed so far for the vertical alignment of LCs involves coating the substrates with suitable alkyl silanes^9^. However, there has been a continuous effort to improve upon the aligning technique for LCs. Several surface modification techniques employed to achieve effective vertical alignment of LCs include formation of self assembled monolayers (SAMs) on substrates, using Langmuir-Blodgett (LB) and Langmuir-Schaeffer (LS) methods, coating polyimide derivates containing hydrophobic long alkyl chain derivatives, irradiating with UV light, doping an organic compound or nanoparticles, or fabricating micro and nanostructures^11–16^. To name few, D. M. Walba *et al*. described the formation of SAMs of trialkoxysilanes on a glass substrate as alignment layers for LC orientation^11^. B. H. Hwang *et al*. reported the successful alignment of LCs with negative dielectric anisotropy on a-SiO_x_ thin film deposited over indium tin oxide (ITO)^17^. In an another example, P. S. Noonan *et al*. demonstrated that, a mixed monolayer composed of long and short chain alkylsilane performed on glass substrates was more effective in bringing about successful homeotropic anchoring of LCs compared to a SAM composed of a single alkylsilane molecule^18^. LC alignment studies over blend polymer surfaces were also reported^19^. Further, a photoswitchable azosilane monolayer on a glass/ITO substrate was also used for the LC alignment^20^. Self-organized thin films of molecules on water surface (Langmuir films) transferred to a solid substrate through Langmuir-Blodgett or Langmuir-Schaeffer techniques were also examined for the effective alignment of LCs. For example, Seki *et al*. demonstrated reversible homeotropic to planar photochemical alignment controls of a nematic LC using photochromic layers comprised of LB films of side chain type azobenzene amphiphilic polymers^13^. E. K. Mann and co-workers have described the perpendicular alignment of a nematic phase exhibited by a bent-core molecule using LS monolayers of BC LCs^21^. In an another approach, K. H. Kim *et al*. described the vertical alignment of LCs on ITO substrate without the alignment layers, simply by mixing the LC with very small quantity (0.05%) of a surface active reagent namely, hexadecyltrimethylammonium bromide^22^. Similarly, an automatic vertical alignment of nematic LCs over an ITO substrate by using carbohydrate-based giant surfactants as alignment layers was reported by D. Y. Kim *et al*.^23^. Following a similar approach, I. Son *et al*. described the vertical alignment of LCs over an ITO substrate by doping the LCs with a small quantity of alkylated benzoic acid derivates which formed a SAM through the attachment of polar acid groups on the substrate surface^24^. Same group have also reported a new strategy for the vertical alignment of LCs wherein, an *in*-*situ* photo-polymerization of alkyl acrylate monomer was carried out in a LC cell resulting in a polymer alignment layer on the ITO substrate^25^. Hwang *et al*. described the characteristics of nanoparticle-doped homeotropic LC device^26^. We also reported an effective homeotropic alignment of bulk LCs over a functionalized ITO substrate^27^. It is interesting to point out here that, most of the approaches described so far for the LC alignment were carried out typically either on glass, indium tin oxide (ITO) or silica thin films and in all these studies, alignment of LC was demonstrated in a LC cell in which the oriented LC sample was confined between two surface modified substrates.

On the other hand, flexible substrates have gained significant attention in recent years, owing to their unprecedented properties such as light-weight, low-cost, thinness, easy processing and popularity of the devices incorporating flexible components since they are more convenient, interesting and easy to store^28–32^. Several plastic materials namely polyethylene terephthalate, polycarbonate, polyurethane, polydimethylsulfoxide etc. have been explored as substrates for different applications^33–37^. As mentioned earlier an effective orientation of LCs is crucial for their applications. However, techniques for LC alignment over flexible substrates are very limited. Some of the proposed methods suffer from drawbacks such as polyimide alignment materials which require higher temperature, photo alignment which lack longtime stability of the alignment direction, vaccum treatment of the composite organic film structure on the plastic substrate which might bring unpredictable deformations and thermal nanoprint lithography which is expensive. Only few examples known so far which describe the LC alignment over a polymer substrate. For example, Sekine *et al*. demonstrated reversible photochemical alignment of a nematic LC using LB films of poly(vinyl alcohal) derivatives containing azobenzene side chains^13^. U. V. Mahilny *et al*. described a polymer-based alignment material named B15 which can be deposited from non-aggressive solution over plastic substrate at low temperature deposition process^38^. Further, H. Kang *et al*. have prepared the poly(methyl methacrylate) derivatives containing cardinal moieties^39^. The polymer was spin coated on ITO coated glass substrates and vertical alignment of LCs was demonstrated in LC cells made by sandwiching two of the polymer film deposited ITO slides using a spacer.

**Figure 1:**
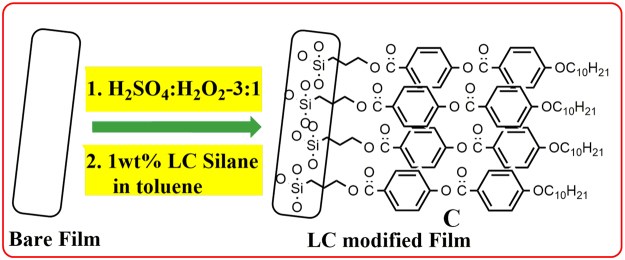
Graphical representation of methodology followed for the surface functionalization of cellulose acetate based flexible polymer film with a LC molecule.

While designing LC alignment layers for plastic substrates, several factors need to be considered such as chemical compatibility, low temperature process, good thermal and chemical stability of the modified substrate, reproducibility and the cost. In this regard, herein, we describe a simple, robust, reproducible technique for the efficient vertical alignment of LCs over an economically viable, easily available flexible polymer substrate having a commercial name, overhead projector (OHP) film (Fig. 1). This film is essentially a transparent plastic film and cellulose acetate is supposed to be the main component of the film. In order to probe the chemical nature of this substrate (elemental composition and presence of functional groups), as-received OHP films were characterized using X-ray photoelectron spectroscopy (XPS) and infrared spectroscopy (IR-ATR mode) techniques (described later) which supported our claim, hence the plastic substrate is referred as cellulose acetate film in the rest of the content. Unlike the other two methods described for LC alignment over flexible polymer^38,39^, in which polymer alignment layer was deposited from a solution over a support (plastic substrate or ITO), our approach involved chemical functionalization of surface of the polymer film in order to create permanent alignment layers on the flexible substrate. Acetate films have remarkable features such as transparency, clarity, high mechanical strength, lightweight, stability under ambient conditions, breakage resistance, smooth finish, low cost and easy availability.

The polymeric substrate was modified with a monolayer of newly synthesized LC compound (**C**) containing a terminal trimethoxysilane moiety in order to obtain an effective LC alignment at the solid substrate – LC interface. The LC molecules chemically attached over flexible substrate were expected to provide necessary shape and symmetry compatibility for the bulk LC sample to be aligned. Characterization of the unmodified and LC modified polymer substrates and versatility of the LC functionalized polymer substrate for the bulk LC alignment are presented. Interestingly, our approach meets several requirements such as ease of the method, surface modification at ambient conditions, good chemical and thermal stability of the modified substrate, long-time stability of the alignment, reproducibility, reusability and low-cost.

## Results and Discussion

Herein, we described a simple, efficient and reproducible approach for the homeotropic alignment of LCs over a cellulose acetate based flexible film at substrate-LC interface. Although appropriate methods for alignment of LCs on different substrates such as glass, silicon wafer and ITO have been investigated in detail, similar studies over emerging, new-generation, interesting substrate material, namely flexible polymer are very limited in number. In this regard, present work describes a promising strategy for effective use of an easily available, cost-effective cellulose acetate based polymeric substrate (commercial name- OHP film), for homeotropic alignment of bulk LCs. The strategy involved the chemical functionalization of surface of the film with a newly synthesized LC compound (**C**) containing a terminal trimethoxysilane moiety through a monolayer formation, which in turn acted as permanent aligning layer for the bulk LC orientation. The motives behind the approach are as follows; chemically attached LC molecules on the surface are expected to provide necessary shape and symmetry compatibility for the bulk LC alignment and the chemical modification of the surface (in comparison to the physical methods) will provide a long term stability and assures reusability of the modified substrates.

**Figure 2:**
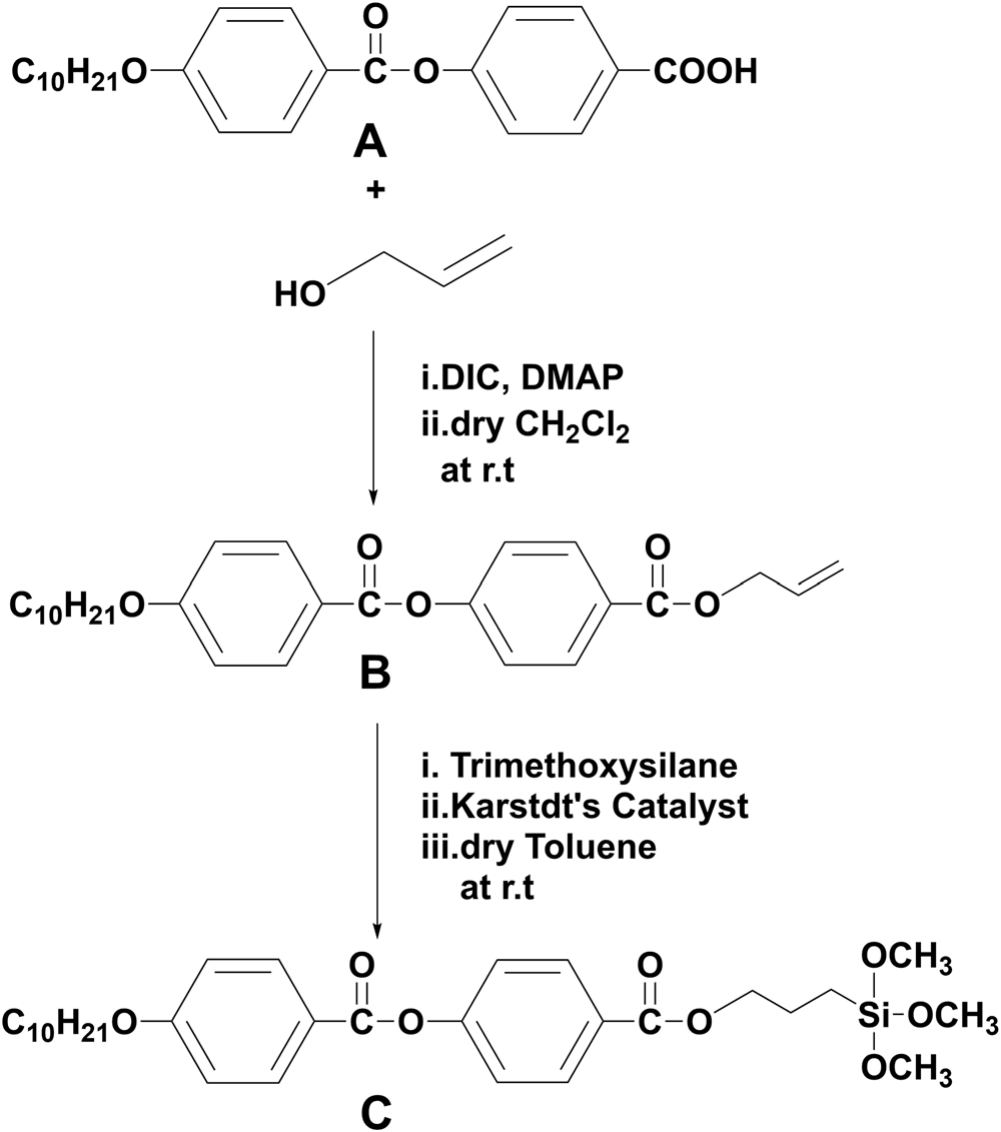
Synthetic route followed to prepare the silane terminated compound **C**.

LC compound (**C**) containing a trimethoxysilane terminal group required for functionalization of the cellulose acetate substrate was synthesized using the starting compound **A** following a pathway shown in Fig. 2 (Synthesis of compound **A** (Fig. S1) along with the analytical data obtained for the compound are provided in supplementary information (**SI**)). The compound **A** was esterified with allyl alcohol resulting in a vinyl terminated compound **B**. In the next step, vinyl moiety in compound **B** was converted to a trimethoxysilane group through a platinum catalyzed hydrosilylation reaction yielding compound **C**.

Mesomorphic properties of the synthesized compounds were investigated using polarizing optical microscopy (POM), differential scanning calorimetry (DSC) and X-ray diffraction (XRD) studies. Both compounds ‘**A**’ and ‘**B**’ were enantiotropic mesomorphic and the phase transition temperatures obtained for these compounds (from DSC thermograms, Figs S5 and S6 respectively SI) are provided in Table S1 (SI). On cooling from the isotropic, a sample of compound **A** sandwiched between a normal glass slide and a cover slip, appeared as droplets which coalesced to form a Schlieren texture with two- and four-brush defects. In addition, a marble texture was also observed in some other areas of the slide. On further cooling, a phase transition was clearly seen at around 180 °C, at which, the two-brush defects in the schlieren texture changed to four-brush defects. These textural features clearly indicated a lower temperature smectic C phase and a higher temperature nematic phase. Recently, we reported LC properties of the two homologues (compounds **1a** and **1b** in ref.^27^) having the same molecular structure as compound **A** but differ in chain lengths (*n* = 8; Cr 141.0 SmC 177.5 N 236.5 I, and *n* = 14; Cr 120.5 SmC 214.5 N 218.0 I)^27^. Textural features exhibited by compound **A** were very similar to that of homologues **1a** and **1b**. A grainy texture observed for smectic C phase of compound **A** at 140 °C and a marble texture obtained for nematic phase at 210 °C are provided in Fig. S7(a) and (b) respectively. It is interesting to notice that transition temperature values of compound **A** (*n* = 10) lie in between that of compound **1a** (*n* = 8) and **1b** (*n* = 14), which is typical for a homologous series.

**Figure 3:**
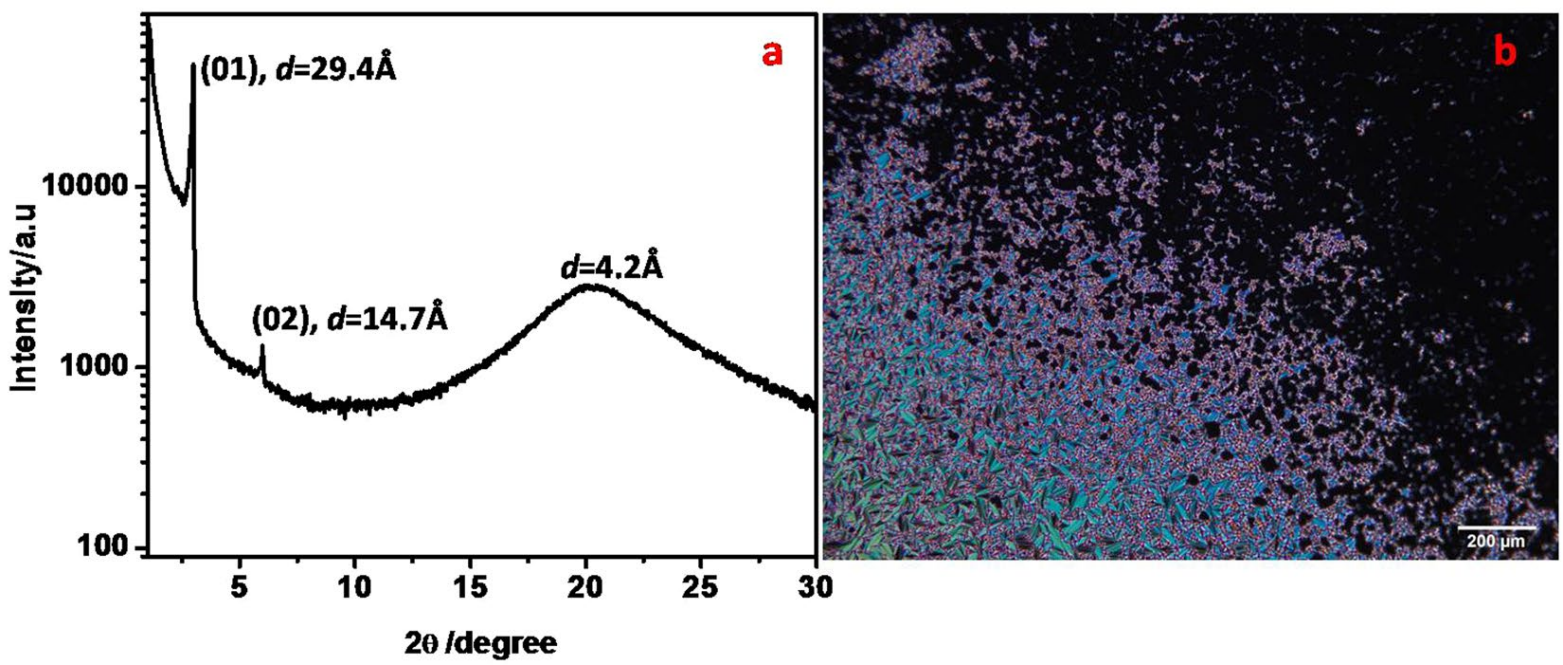
(**a**) X-ray diffractogram obtained for mesogen **B** at 45 °C (on cooling from isotropic) showing periodic reflections. (**b**) POM image of compound **B** between a glass slide and a cover slip at 62 °C.

On the other hand, compound **B**, exhibited a single phase with a thermal range of 16.3 °C in heating cycle and a wider thermal range of 39.3 °C in the cooling cycle, as indicated by DSC (Fig. S6) and POM studies. Upon viewing under POM, a sample of **B** between a glass slide and a cover slip, displayed a focal-conic texture. In addition to this texture, a homeotropic texture was also observed in some regions of the sample. These textural features indicated the presence of a smectic A (SmA) phase and this phase assignment was further confirmed through XRD studies. A sample of mesogen **B** showed two reflections in the small angle region having the ‘*d*’ spacing values of 29.4 Å and 14.7 Å. These layer spacings are in the ratio 1:1/2 suggesting a smectic periodicity in the mesophase. Further, the first order layer spacing calculated from XRD data (29.4 Å), matched well with the measured molecular length of the mesogen (29.1 Å), considering all-*trans* extended conformation of the terminal alkyl chain (ACD/chemsketch-3D viewer freeware). This confirmed that molecules lie with their long axis (director) parallel to the layer normal in the mesophase and hence the presence of smectic A phase. Additionally, a diffused reflection was observed in the XRD pattern, at around 4.3 A° in the wide angle region indicating the fluidity of the mesophase. An X-ray angular intensity profile obtained for compound **B** in the mesophase and a texture displaying both focal conic and homeotropic regions are provided in Fig. 3(a) and (b) respectively.

Compound **C** also displayed the textural features of SmA phase (Fig. S8), however with broad transition temperatures. Compound **C** having a terminal trimethoxysilane moiety was employed for surface modification of flexible substrate. Confirmation of functionalization of the flexible film with LC molecules was obtained by XPS, ATR-IR, CA and AFM studies.

**Figure 4:**
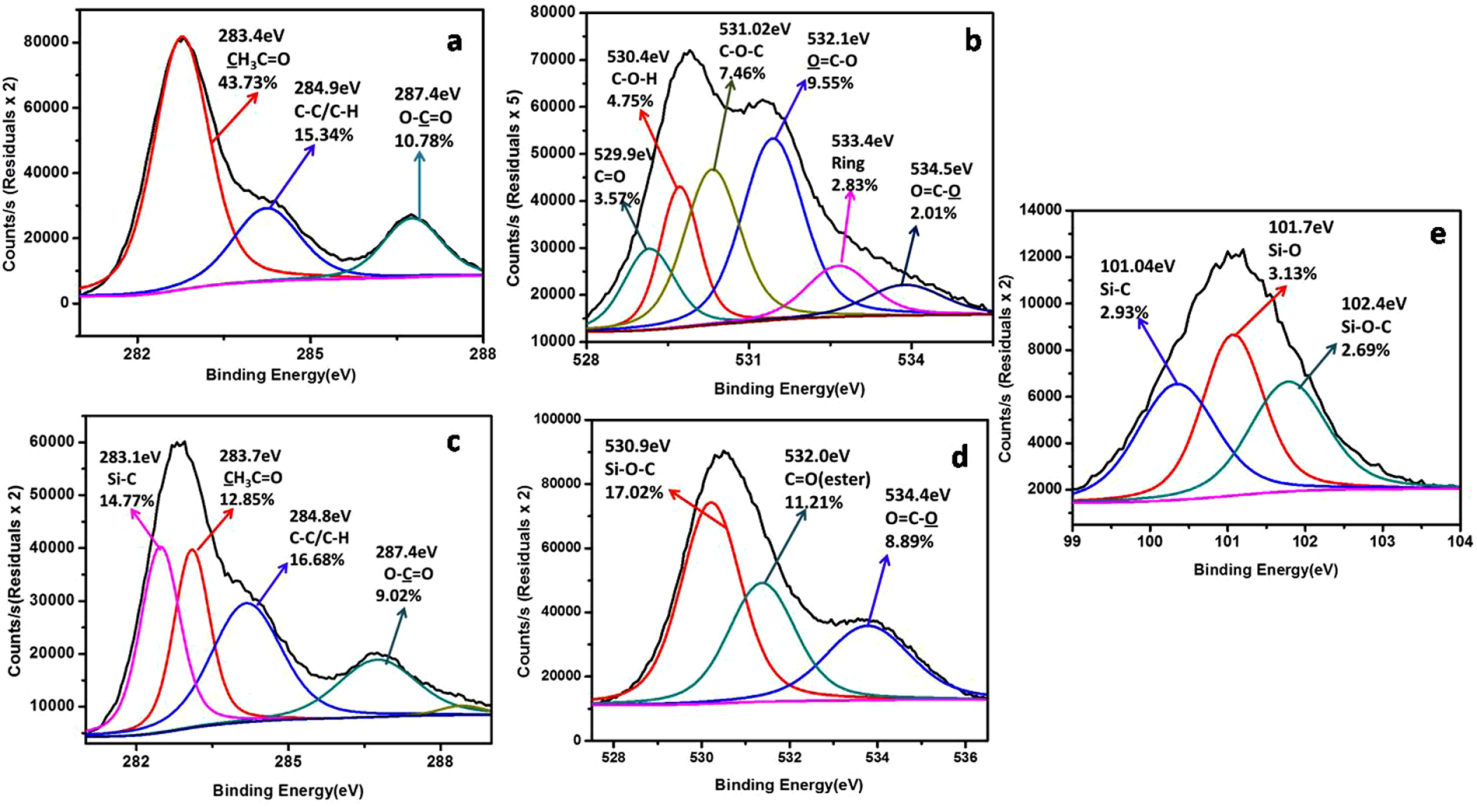
(**a**,**b**) C1s and O 1s XPS spectra for unmodified substrate and (**c**–**e**) C 1s, O 1s and Si 2p XPS spectra for surface modified substrate respectively.

XPS analysis was used to identify the chemical nature of the flexible substrate before and after surface modification. XPS spectra obtained for as-received(unmodified) and modified substrate (individual scans for different elements observed) are provided in Fig. 4 (elemental survey scan SI, Fig. S10). XPS spectrum of unmodified substrate showed the presence peaks corresponding to C 1s and O 1s (Fig. S10a). Surface modified film also displayed peaks corresponding to C 1s and O 1s. However, in addition the spectrum exhibited peaks due to Si 2 s and Si 2p (from the terminal silane moiety of LC), thus confirming the immobilization of LC molecules (Fig. S10b). The C 1s, O 1s spectra for unmodified substrate are provided in Fig. 4a and b whereas the corresponding C 1s, O 1s and Si 2p spectra for surface modified substrate are provided in Fig. 4c–e respectively. For the unmodified film, the observed C 1s binding energies were assigned as follows: the peak at 283.4 eV is due to methyl carbon of the acetyl groups [H_3_**C**(C=O)], 284.9 eV due to the carbon atom in C-C/C-H moieties and at 287.4 eV which is attributed to carbon atom in O-C=O and O-C-O moieties^40^. Similarly the O 1 s binding energies displayed by the untreated substrate at 529.9, 530.4, 531.02, 532.1, 533.4, 534.5 eV were assigned to a ketone [C-C(O)-C], hydroxyl group O-H, ether (C-O-C), ester carbonyl (RC**O**O), oxygen in a ring^41^ and oxygen next to a carbonyl group in an ester (-CO**O**)^42,43^. The presence of these functional groups, especially BE’s corresponding to a methyl carbon of acetyl group and a ring oxygen (may be assigned to a pyranose ring) clearly pointed towards the presence of cellulose acetate as the main component of our flexible substrate.

Similarly, surface modified substrate displayed C 1s peaks at 283.7, 284.8, 287.4 eV corresponding to [H_3_**C**(C=O)O], C-C/C-H, O-C=O moieties respectively. Additionally, a peak was observed at 283.1 eV which is attributed to Si-C bond in the LC molecules^20^. It is interesting to notice here that, the peak intensity of C 1 s peak at 283. 1 eV corresponding to [H_3_C(C=O)] is significantly reduced (43.73% to 12.85%) from bare to modified substrate which indicated the possible hydrolysis of acetate ester resulting in removal of acetyl groups during surface treatment. Besides, the O 1 s XPS spectrum of modified substrate exhibited peaks at 530.9, 532.0 and 534.4 eV which are assigned to Si-O-C, O-C=**O** and **O**-C=O moieties. Furthermore the Si 2p spectrum displayed peaks at 101.04, 101.7 and 102.4 eV which are due to Si-C, Si-O and Si-O-C functional groups^44^. Thus, the XPS results indicated the presence of cellulose acetate in the substrate and provided evidence for the surface functionalization of the cellulose acetate substrate with LC molecules.

**Figure 5:**
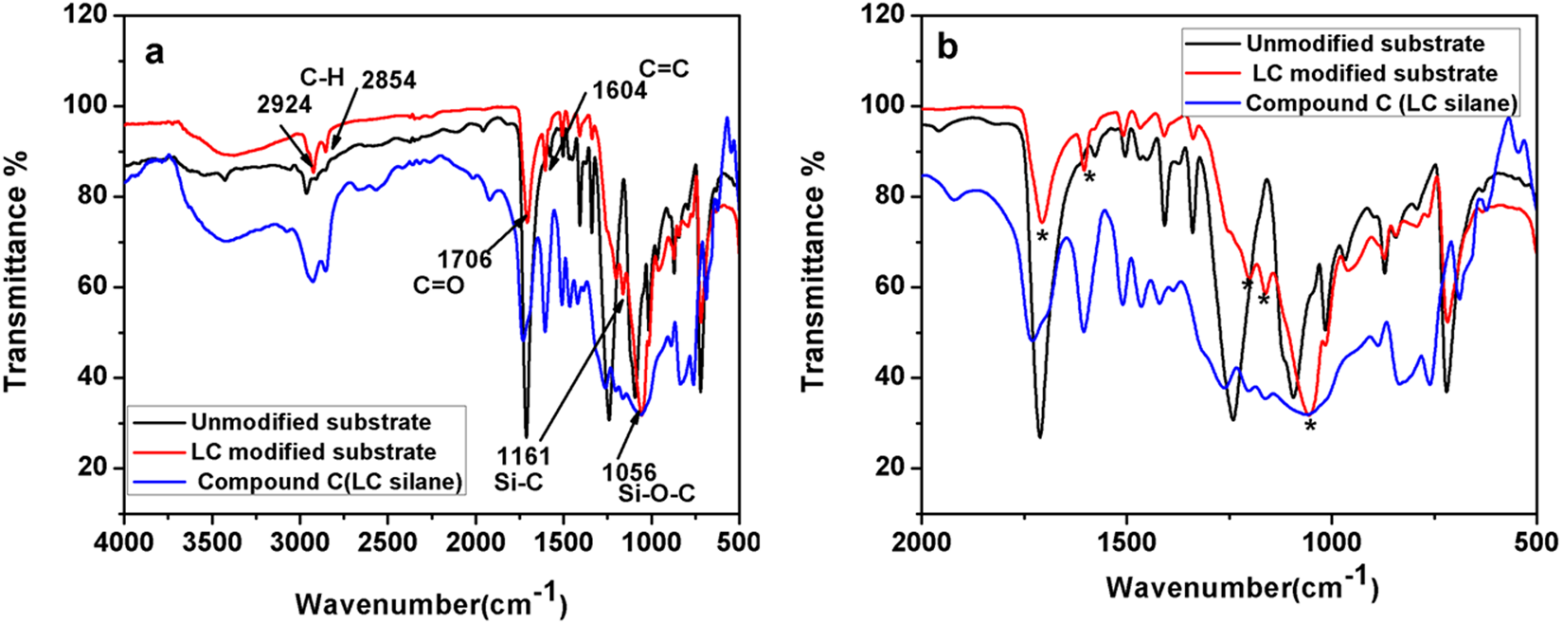
ATR**-**FTIR spectra recorded for (**a**) bare cellulose acetate film, LC modified film and LC silane compound. (**b**) Expanded version of the graph shown in (**a**) for the region 2000–400 cm^−1^.

Attenuated total reflection IR (ATR-FTIR) studies further supported the presence of cellulose acetate in our substrate and confirmed the surface functionalization of the this substrate with LC molecules. An overlap of IR spectrum of the bare film before and after chemical modification along with spectrum of silane terminated LC molecule, **C** (for comparison), is provided in Fig. 5. IR spectrum of as-received flexible polymer film displayed prominent bands at 2963 cm^−1^ and 1712 cm^−1^ which correspond to stretching vibration of CH_2_ groups and stretching modes of ester -C=O respectively. Sharp bands observed at 1241 cm^−1^ and at 1093 cm^−1^ can be attributed to -C-O single bond stretching modes. These peaks (1241 cm^−1^ and 1093 cm^−1^) can be associated with the carboxylate group, ether link between rings C-O-C and the pyranose ring, respectively^45,46^. In addition, the polymer film also displayed medium intensity bands 1407 and 1339 cm^−1^ which can be attributed to bending and rocking vibrations of CH_2_ groups, respectively. All these details from the IR spectrum supported the presence of cellulose acetate component in the flexible plastic substrate. Therefore, based on XPS and IR analysis we claim that main chemical component our flexible substrate is cellulose acetate (besides the plastic substrate material might also contain a plasticizer to improve the flexibility and durability).

The spectral pattern of modified substrate differed from that of the bare (unmodified) suggesting a successful surface functionalization. However, few characteristic bands of unmodified substrate were retained intact (1015, 967 and 720 cm^−1^ in the finger print region), even after surface modification. Moreover, it can be clearly seen from the figure that IR spectrum of the modified substrate follows the spectral pattern of LC-silane along with strong bands at 1056 cm^−1^ and 1162 cm^−1^ which can be attributed to Si-O-C and Si-C (in Si-CH_2_) stretching vibration (these peaks are absent in unmodified film). Incidentally, similar IR bands at almost same wave numbers have been reported for silane condensation on other substrates^47,48^. In addition, modified film also displayed vibrational bands at 2924, 2854 cm^−1^ which correspond to antisymmetric and symmetric stretching of CH_2_ groups, at 1706 and 1201 cm^−1^ which can be attributed to -C=O and C-O stretching modes and also a sharp band at 1604 cm^−1^ which is due to the C-C stretch in the aromatic ring. Interestingly, the corresponding peaks with nearly matching wavenumbers can be seen in the spectrum of LC silane. (The broad band at around 3400 cm^−1^ (O-H stretching vibrations) observed in spectrum of both silane compound and surface modified film are due to moisture from the ambience). These details clearly indicated the surface functionalization of the substrate with LC molecules.

Thus, IR investigations corroborated the observations made using XPS studies and together these techniques confirmed the successful surface modification of cellulose acetate substrate with LC molecules.

**Figure 6:**
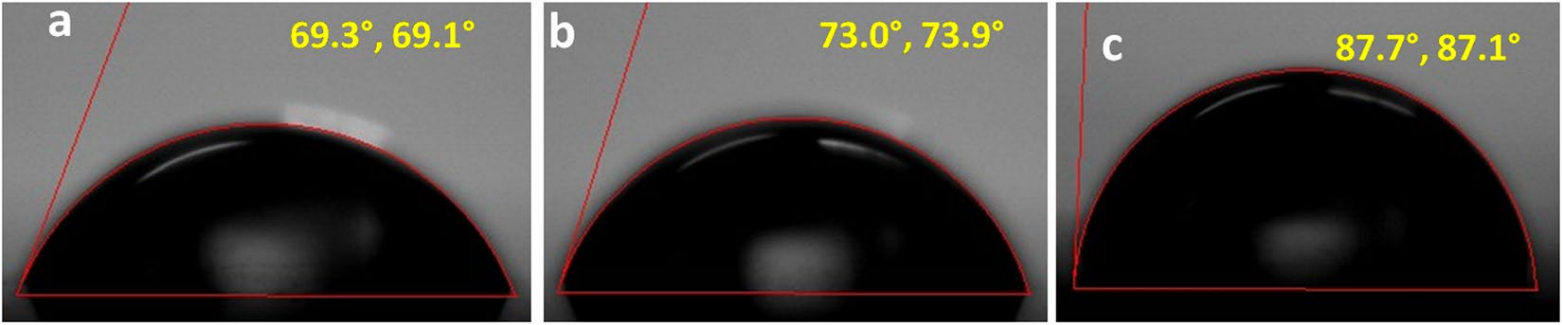
Static water CA images recorded for (**a**) as received (before modification), (**b**) pre-treated and (c) chemically functionalized cellulose acetate substrates.

Further, CA measurements were carried out to study the surface wettability of the bare, pre-treated and LC modified substrates using water sessile drop method. An average CA value of 69 ± 2° was measured for as-received substrate indicating a hydrophilic surface. Similar water CA values are reported for cellulose acetate film in the literature^49^. On the other hand, an average CA value of 73.1 ± 1° was recorded for pre-treated substrate. Generally, a reduced value of CA is expected after pre-treatment, since piranha cleaning is supposed to introduce surface hydroxyl groups (hydrolysis of cellulose acetate). Cellulose acetate already contains hydroxyl groups and after piranha cleaning the no. of free hydroxyl groups are expected to increase. However, CA values also depend on the surface roughness in addition to the nature of the surface (hydrophilic or hydrophobic). A smoother surface is supposed to produce a lower CA value compared to a rough surface. An increased value of CA observed for pre-treated substrate indicates that piranha cleaning might result in a roughened surface. Our speculation is supported by AFM observation (described later). The CA value significantly increased for LC modified substrate (86.2 ± 1°). This increased value of CA measured for modified polymer film thus confirmed the attachment of LC molecules (with terminal alkoxy chains extending out on the surface) on the flexible substrate resulting in a hydrophobic surface. Images of the water static CA recorded for bare (unmodified), pre-treated and LC modified films are shown in Fig. 6(a–c) respectively.

**Figure 7:**
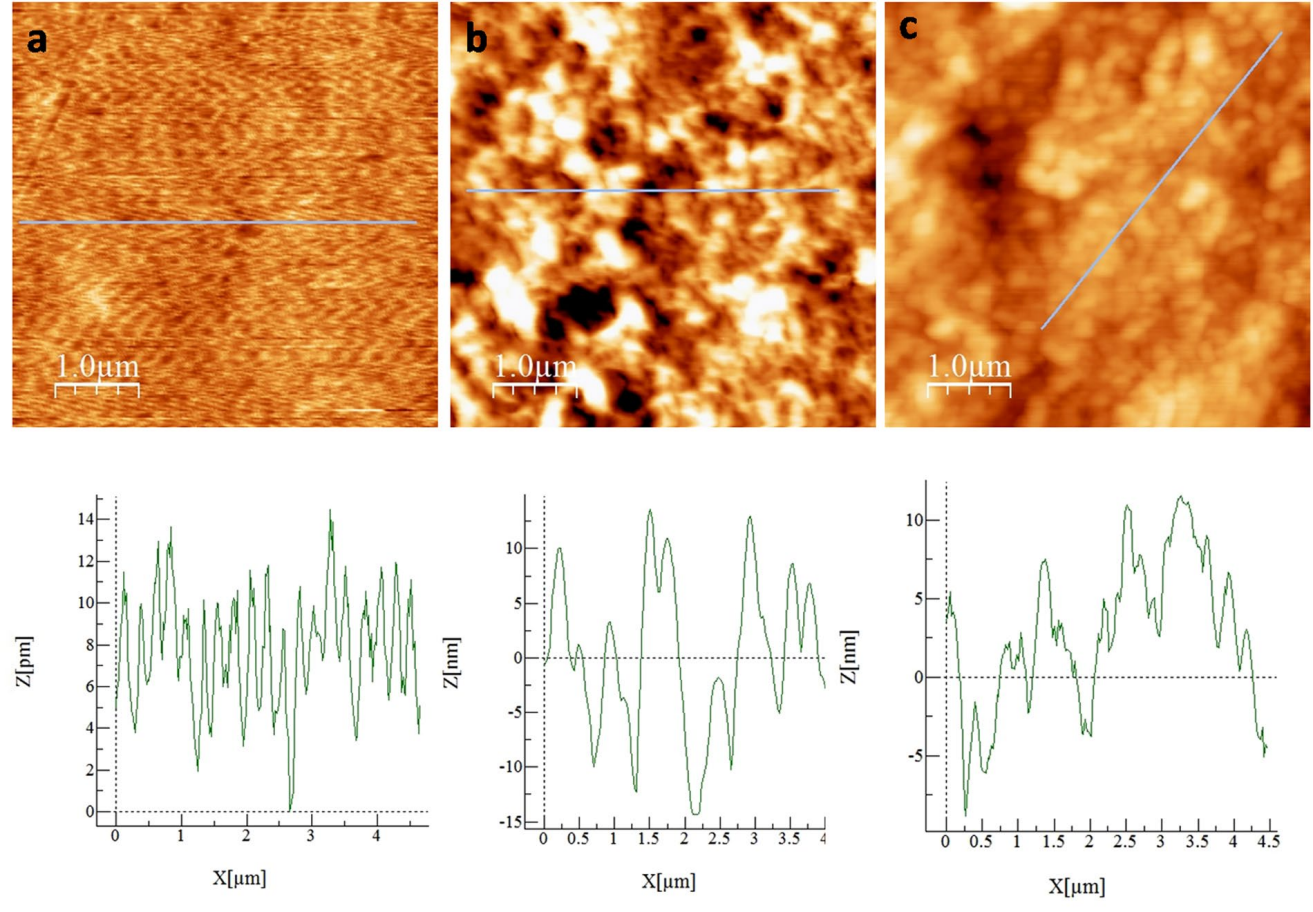
AFM images (1 µm × 1 µm) and the corresponding line profiles (recorded along the straight lines shown in respective images) recorded for (**a**) cellulose acetate substrate before modification, (**b**) pre-treated and (**c**) LC silane modified substrate.

In addition, AFM studies were carried out to investigate the change in surface morphology before and after chemical modification of the flexible substrate. Representative AFM images (1 μm × 1 μm) obtained for bare (before modification), pre-treated and LC modified substrates along with their corresponding line profiles are shown in Fig. 7a–c respectively. The unmodified substrate displayed a root-mean-square (rms, R_q_) roughness value of 2.62 nm. However, the roughness factor value of pre-treated substrate is calculated to be 7.0 nm as a result of change in the surface morphology. Interestingly after the immobilization of LC, this roughness value is increased further to 8.4 nm. Formation of grain boundaries observed in the AFM image (Fig. 7c) account for the increased roughness which indicated the presence of probable defects in the monolayer arising out of immobilization of LC. The CA value obtained for LC modified substrate (∼87°) is lower than the CA value generally observed for a well packed defect free monolayer (CA > 100°), supports AFM observations. However, we speculate that the defects in the monolayer might be beneficial for a perfect bulk LC alignment by allowing the infiltration of LC molecules to the self assembled monolayer environment, where they interact with LC molecules attached to the substrate so that they (bulk LC) align perpendicular to the interface. Previous reports also have demonstrated that mixed monolayer of alkyl silanes containing long and short hydrocarbon chains is more effective in inducing vertical alignment of LCs compared to a dense, compact monolayer of a single long alkyl chain compound^18,50^.

Following the confirmation of successful immobilization of LC molecules over cellulose acetate substrate, the modified films were assessed for their ability to orient the bulk LC sample. For this purpose, a well-known mesogen, 4′-pentlybiphenyl-4-carbonitrile, commonly known as **5 CB**, which exhibits a room temperature nematic (5CB; Cr 24 N 35.5 I) phase and mesogen **B** (precursor to the LC silane molecules attached on the polymeric surface) which shows a smectic A (SmA) phase were chosen as analytes. POM is a simple, effective tool to study orientation of molecules in a particular mesophase with respect to the substrate surface. A complete dark texture is observed under POM with crossed polarizers, when the director (average local direction of the long molecular axis) of a mesophase is oriented perpendicular to the substrate (homeotropic anchoring). In this director configuration, optic axis of the system is parallel to the direction of light propagation and hence, there is no birefringence for any in-plane rotational position of the sample between crossed polarizer. For comparison, LC alignment studies were also performed on polymer substrates treated with octadecyltrimethoxysilane (OTS), a commonly used reagent for LC alignment.

**Figure 8:**
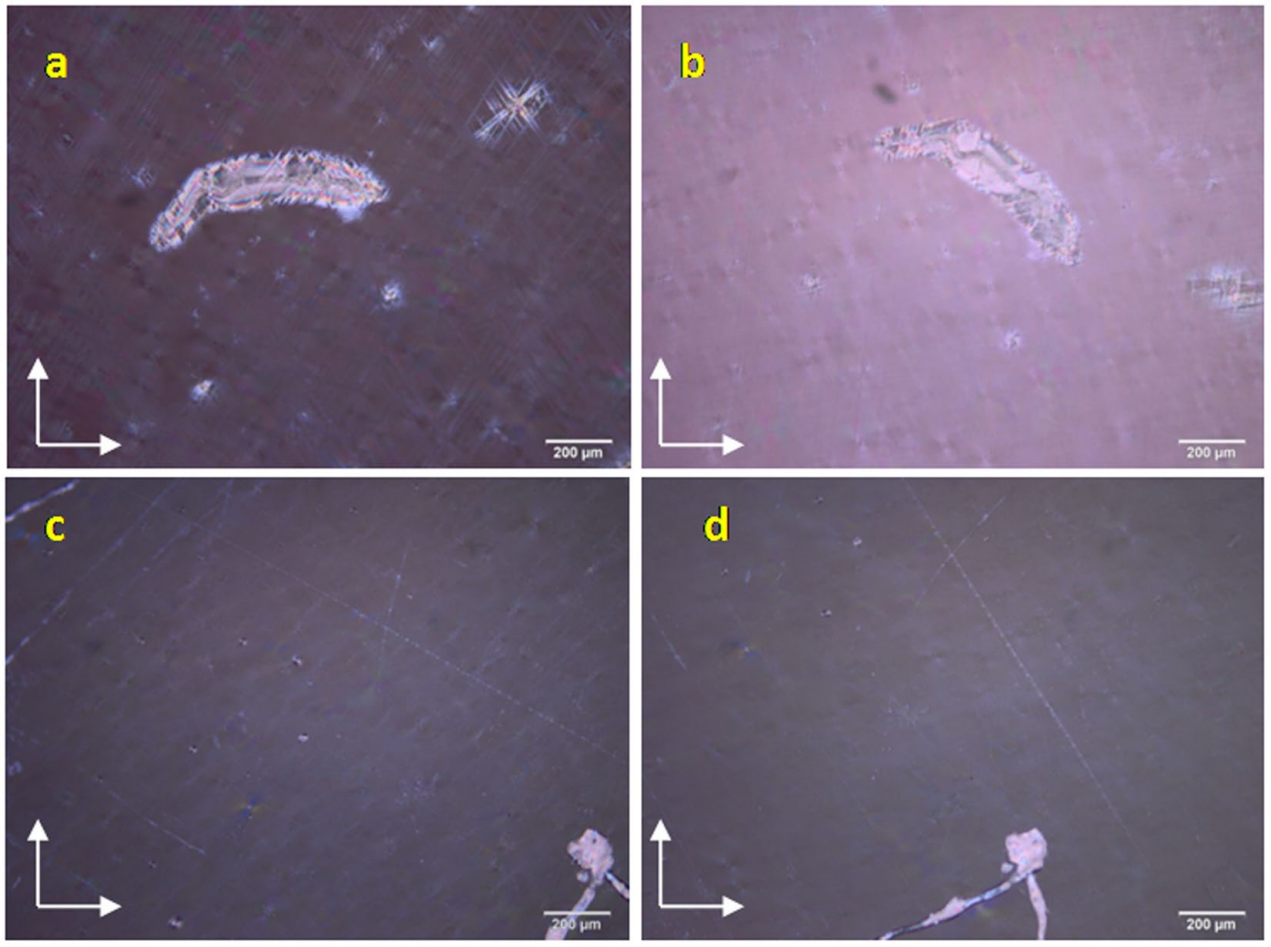
POM images of 5CB at 25 °C recorded over OTS coated substrate (**a** and **b**) and LC functionalized substrate (**c** and **d**). Arrows in the image indicate the direction of polariser and analyser. Images on the left are rotated through an angle of 45° from the original position.

A thin film of LC sample was formed on OTS coated and LC modified polymer films by drop-casting a solution of respective LC (1 wt%) in dichloromethane (2 µl), dried at ambient conditions and alignment of the LC director over the substrates was studied under a POM. In order to distinguish a perfect homeotropic alignment from a tilted orientation of the LC molecules, POM images were also recorded by rotating the sample stage.  Fig. 8 shows POM images of N phase on OTS coated (8a and b) and LC functionalized substrates (8c and d). A mixture of dark and birefringent region was observed for the N phase on OTS treated surface indicating a partly aligned orientation of the nematic director. However, a complete dark texture was observed for the mesophase over LC modified substrate, indicating a uniform perpendicular orientation of the director with respect to the substrate plane. A similar complete dark texture was observed for the nematic phase throughout the sample surface of LC modified substrate indicating a perfect uniform homeotropic alignment of the nematic director over this surface (Movie S1). Analogous behavior was also observed for the samples in which 5CB was drop-casted neat over the modified substrate. Further, a change in transmitted light intensity was noted for nematic phase over OTS treated substrate upon rotating the stage to 45° from the original position (Fig. 8b) whereas the light intensity remained same for the N phase over LC modified substrate (Fig. 8d) upon rotating the sample stage to 45° thus confirming vertical alignment of nematic director over LC modified substrate.

**Figure 9:**
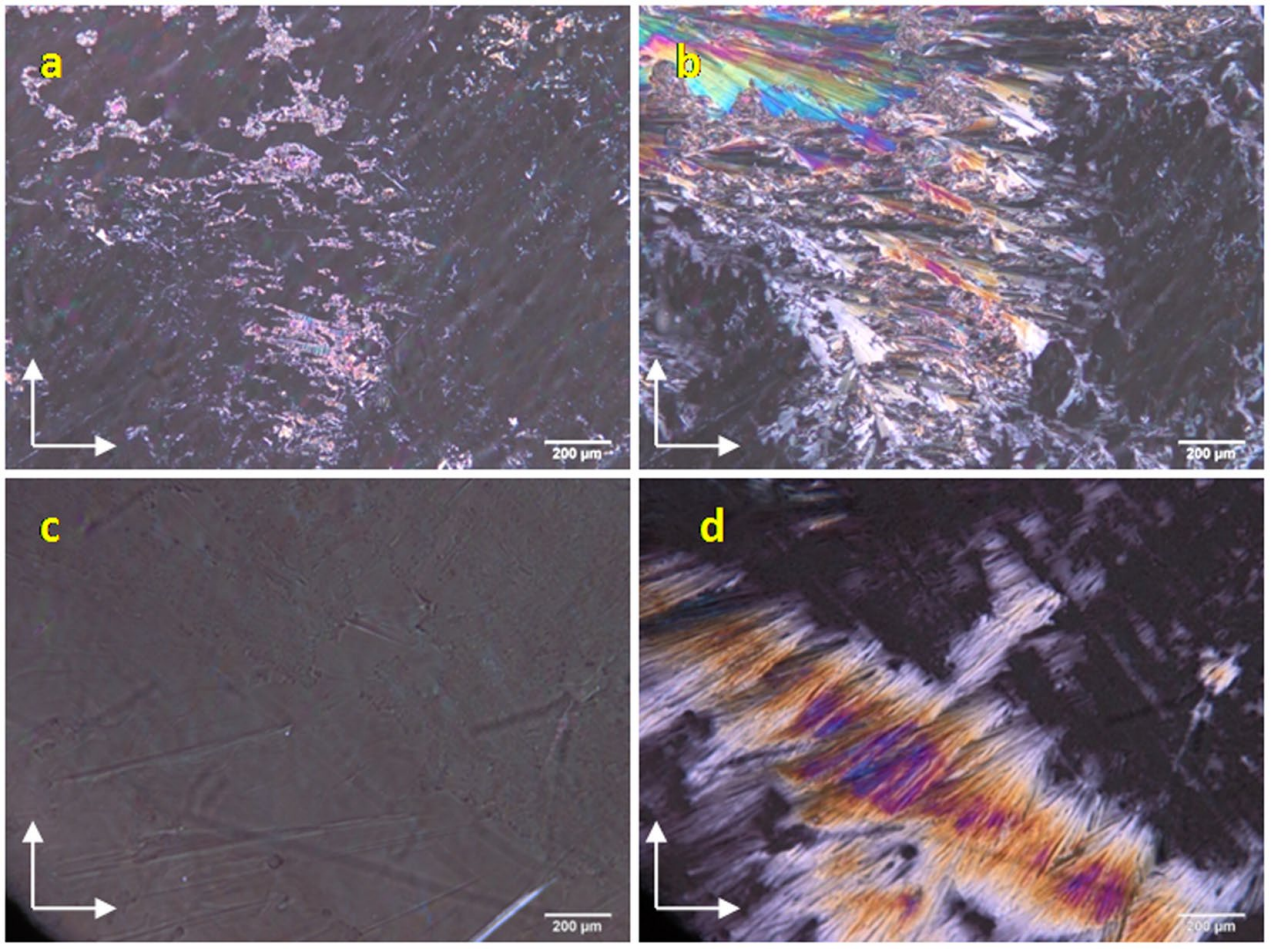
POM images of mesogen **B** over OTS modified (**a** and **b**) and LC modified cellulose acetate substrates (**c** and **d**). Images on the left are at 60 °C showing the texture of SmA phase; images on the right are recorded at 35 °C displaying the texture of crystalline phase;. Arrows indicate the direction of polariser and analyser.

On the other hand, substrates containing the LC sample **B** were heated in a hot stage and textures were observed under POM. Prior to these experiments, cellulose acetate polymer substrates were heated to 80 °C for 18 hrs and were found be intact after heat treatment. No change in the physical appearance was observed (such as softening of the film) and chemical nature was also found be unaltered as observed from IR. POM images of SmA and crystalline phase (obtained on cooling from isotropic) over OTS coated substrate and LC modified substrate are provided in Fig. 9(a–d) respectively.

**Figure 10:**
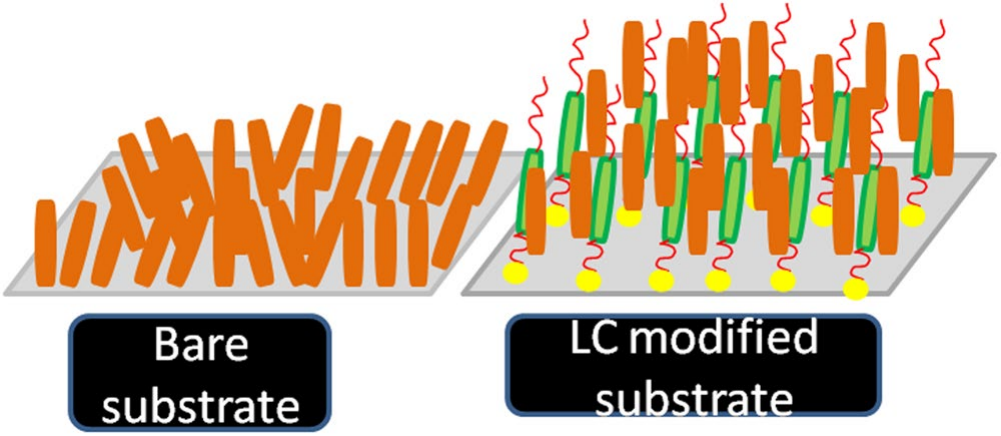
Schematic representation of orientation of bulk LC sample over bare and LC modified cellulose acetate films.

On cooling from the isotropic, sample **B** over OTS modified cellulose acetate polymer substrate displayed a dark texture indicating a vertical director configuration with respect to substrate, few birefringent regions could still be seen suggesting that, the perpendicular orientation of the director with respect to the substrate plane is not uniform (Fig. 9a) over entire area of the substrate. On contrary, LC sample over LC modified cellulose acetate film exhibited a complete dark texture upon phase transition to smectic A phase, thus indicating an effective vertical alignment of the director (Fig. 9c). Infact, such vertical alignment of the director existed over entire region of the sample surface, depicted through the appearance of complete dark texture under POM. These observations clearly demonstrated that, although OTS is well known for inducing vertical alignment of LCs over glass, ITO and other substrates, it was found to be not very effective in aligning LCs over cellulose acetate containing flexible polymer substrate. In comparison to OTS treated cellulose acetate based flexible substrate in which silane molecules are physiadsorbed on the substrate, LC modified cellulose acetate polymer substrate is highly efficient in offering perfect homeotropic boundary conditions for the effective bulk LC alignment. This in turn prove that the LC layer with an extended alkyl chains over the substrate provides a necessary compatibility for the bulk LC sample, resulting in a effective homeotropic alignment of bulk LCs (Fig. 10).

In addition to be used as a single substrate for vertical alignment of LCs at a solid-LC interface, these chemically modified substrates were also useful in orienting the LC director in LC cells made by sandwiching two of the modified substrates using a spacer. Alignment of 5CB in LC cells made by sandwiching two bare and two modified cellulose acetate plastic substrates separated by a 6 µm spacer, is shown in Fig. S11a and b respectively. A dark texture under crossed polarizers was observed for the nematic phase in LC cell composed of LC modified polymer substrates (Fig. S11b) on contrary to an uneven texture observed in LC cell made up of untreated films. This observation clearly emphasizes the versatility of the LC modified cellulose acetate substrates for the uniform alignment of bulk LC sample.

It is interesting to point out here that generally it is difficult to apply silane-based alignment compounds on some polymeric substrates such as SU8 (epoxy-based negative photoresist commonly used as structural material in lithographic fabrication), since oxidation of the polymer substrate does not result in silanol (-Si-OH) groups which can further react with the silane compound resulting in strong Si-O-Si bond. Commonly, oxygen plasma treatment and chemical silanization (bifunctional molecule such aminopropyltrimethoxysilane) are used to bond polymer SU8 with polydimethylsiloxane (PDMS) to create microfluidic channels^51^. The surface characterization of our plastic substrate (XPS and ATR-IR) indicated the presence of cellulose acetate. We propose that, the acetyl groups in the polymer were hydrolysed during piranha treatment (using a mixture of H_2_SO_4_ and H_2_O_2_ −3:1 v/v ratio) yielding surface hydroxyl groups which were subsequently reacted with trimethoxysilane terminated LC molecules resulting in Si-O-C linkage and immobilization of the LC molecules on the surface. This Si-O-C linkage was found be stable in our substrate for common solvent and thermal treatment. Reproducible contact angle and IR (ATR mode) results obtained for the LC modified substrate before and after subjected to solvent and thermal treatment confirmed the stability of the Si-O-C in our substrate. It is worth mentioning that hydrolytic stability of the Si-O-C linkage in some of the copolymers and formation of stable Si-O-C submonolayers on silicon surface has been reported in literature^52,53^.

Overall, we have shown that a cellulose acetate based flexible polymer film can be surface modified with a LC compound containing a terminal silane moiety in a simple approach. The LC functionalization of the polymer substrate was confirmed through XPS, IR, CA and AFM studies. LC compound modified transparent cellulose acetate substrates are highly efficient in aligning the bulk LC sample. These LC modified polymeric film is stable for several months (more than 12 months) and retains its aligning ability intact. Also, it is interesting to point out here that, the chemically modified LC films are thermally stable and chemically resistant to solvent treatment. More importantly, the modified films are reusable simply by washing off the bulk LC sample from the surface and reused films showed maximum aligning efficiency. These LC modified flexible transparent substrates, low-cost light-weight materials with the ability to align the bulk LC sample have significant applications in LC based sensing and to study biomolecular interactions. Moreover, our technique can be applied to align other class of interesting LCs such as discotics and bent-core compounds, the work is currently under progress. Considering the enormous amount of focus towards the flexible substrates in recent years^28–33^, the described method will pave a pathway for utilization of flexible substrates for various LC based applications in a broad range.

## Conclusions

An uniform homeotropic orientation of LCs at a solid –LC interface over a transparent flexible substrate containing cellulose acetate is demonstrated for two LC phases (nematic and SmA), through chemically functionalizing the film by a newly synthesized LC compound. More interestingly, these LC functionalized polymer substrates are stable over a long period of time, retain their aligning ability and they are reusable with maximum efficiency. In addition to be used as a single substrate for vertical alignment of LCs at a solid-LC interface, these chemically modified substrates are also useful in vertically aligning the LC director in LC cells made by sandwiching two of the LC modified substrates using a spacer.

## Experimental

### Materials

4-(Decyloxy)benzoic acid, benzyl-4-hydroxy benzoate, *N*,*N*′-diisopropylcarbodiimide (DIC), 4-(dimethylamino)pyridine (DMAP), 10% Pd-C, trimethoxysilane, karstedt’s catalyst (platinum (0)-1,3 divinyl-1,1,3,3-tetramethyldisiloxane complex solution in xylene) were purchased from Sigma Aldrich and used without further purification. The solvents used in the synthesis namely, dichloromethane, 1,4-cyclohexadiene, allyl alcohol, toluene, were of analytical grade. As received solvents were dried over molecular sieves (4 Å, 1–2 mm from Alfa Aesar) and the resulting anhydrous solvents were used to carry out the reactions. All the intermediate compounds were purified by column chromatography on silica gel (60–120 mesh) followed by repeated crystallization.

### Synthesis

#### Synthesis of allyl4-((4-decyloxy)benzoyloxy)benzoate, B

4-((4-decyloxy)benzoyloxy)benzoic acid (1 g, 2.5 mmol), **A**, prop-2-en-1-ol (0.1452 g, 2.5 mmol) and DMAP (0.030 g, 0.25 mmol) were dissolved in dry dichloromethane (15 ml) and the resulting solution was stirred for 15 minutes. Following this, DCC (0.418 ml, 2.7 mmol) was added and stirring continued for an overnight at r.t. Thereafter, solvent from the reaction mixture was evaporated and the crude product obtained was purified through column chromatography on silica gel (60–120 mesh) by using hexane/ethyl acetate mixture (9:1) as an eluent. Removal of the solvent from the eluate provided a white product. Yield: 80%, m.p. 54 °C. Analysis: Proton nuclear magnetic resonance spectroscopy (^**1**^**H NMR)** (400 MHz, CDCl_3_) δ (ppm): 8.1–8.3 (m, 4H, Ar-**H**), 7.3–7.5 (d, 2H, Ar-**H**), 6.8–7.0 (d, 2H, Ar-**H**), 6.0–6.1 (m, 1H, -CH_2_-C**H**=CH_2_), 5.2–5.3 (d, 1H, -CH=C**H**_2_ trans proton), 5.3–5.5 (d, 2H, -CH=C**H**_2_ cis proton), 4.7–4.9 (d, 2H, Ar-O-**CH**_**2**_-CH=CH_2_), 4.0–4.2 (t, 2H, -CH_2_-C**H**_**2**_-O-Ar), 1.7–1.9 (quin, 2H, -CH_2_-C**H**_**2**_-CH_3_), 1.3–1.5 (m, 14H, -(**CH**_**2**_**)**_7_-**)**, 0.7–0.9 (t, 3H, -CH_2_-C**H**_**3**_). Fourier transform infrared spectroscopy (**FTIR)** (KBr) ʋ_max_: 3077, 2921, 2853, 1723, 1606, 1509, 1209, 1107, 884, 844 cm^−1^.

#### Synthesis of 4-[(3-(trimethylsilyl)propoxy)carbonyl]phenyl 4-(decyloxy)benzoate, C

Compound **B** (0.5 g, 1.1 mmol) was dissolved in dry toluene (10 ml) and trimethoxysilane (700 µl, 5.5 mmol) was added under nitrogen atmosphere and stirred for 30 min. To this solution, Karstedt’s catalyst (25 µl, 0.11 mmol) was added and stirring continued under nitrogen atmosphere for further 18 hours. Subsequently, the solvent and excess trimethoxysilane were removed under vacuum resulting in a brown residue. This product was used directly for SAM formation without any further purification, since our attempts to purify the silane using chromatography were unsuccessful. However, the residue obtained was dissolved in dichloromethane and filtered using syringe filters to remove the insoluble impurities. The solvent from the filtrate was evaporated and the solid product obtained was dried thoroughly under vacuum and used for IR and NMR characterization. Yield: 85% Analysis: ^**1**^**H NMR**(400 MHz, CDCl3) δ (ppm): 8.07(m, 4H, Ar-**H**), 7.2(d, 2H, Ar-**H**), 6.9(d, 2H, Ar-**H**), 4.2(t, 2H, Ar-COO-C**H**_**2**_), 3.9–4.1(t, 2H, Ar-O-C**H**_**2**_), 3.5(s, 9H, -Si-(**OCH**_**3**_**)**_**3**_, 1.7(m, 2H, Si(OCH_3_)_3_-CH_2_-**CH**_**2**_), 1.2–1.4(m, 14H, -(**CH**_**2**_-)_7_, 0.8–0.9(t, 3H, -CH_2_-**CH**_**3**_), 0.5–0.7(t, 2H, -Si-**CH**_**2**_), **FTIR** (KBr) ʋ_max_: 3076, 2926, 2853, 1729, 1604, 1262, 1202, 1057, 887, 834 cm^−1^.

After the hydrosilylation reaction, compound ‘**C’** was not isolated (attempts to purify the silane either through chromatography or by crystallization were unsuccessful) but used directly from the reaction mixture for the surface modification of substrate. However, the presence of **C** in the residue (obtained after evaporating the solvent from the reaction mixture) was confirmed through IR and NMR studies. IR spectrum of the residue showed all the bands characteristics of alkene-terminated mesogen **B**, along with additional bands at 1057 cm^−1^ and 1262 cm^−1^, corresponding to the stretching vibrations of Si-O-C and Si-CH_2_ groups. In addition, NMR of the sample (Fig. S4) showed no peaks in the region 5.0–6.5 ppm clearly indicating the absence of alkene (-C**H**=C**H**_**2**_) protons and exhibited a characteristic multiplet at around 0.7 ppm [-C**H**_**2**_-Si-(OCH_3_)_3_] and a strong singlet at 3.5 ppm [(-Si-(**OCH**_**3**_**)**_**3**_)], confirming the presence of silane compound.

#### LC functionalization of polymer substrate

Commercially available polyester OHP films (A4size, 100 μm) were purchased from a local shop. As-received sheet was cut into strips of 1 cm (b) ×5 cm (l) dimension and 2 to 3 of these strips were initially cleaned using acetone and water in sequence through sonication and dried under a stream of nitrogen gas. Then the films were rinsed with a freshly prepared piranha solution (H_2_SO_4_ and H_2_O_2_ in 3:1 v/v ratio) for about 2–3 minutes. Following this, the polymer strips were washed with excess amount water, sonicated in a mixture of ethanol and water for about 15 minutes and dried at room temperature for 5–6 hours. Treatment with piranha solution is supposed to etch the surface and these etched polyester films were subsequently dipped in LC silane solution (1 wt% of LC silane in toluene) for an overnight for surface coating of LCs. Then the films were cut into small pieces of pre-defined geometric area (1.5 cm × 2 cm) and used for surface characterization through IR, AFM and CA studies and subsequent LC alignment studies.

#### Octadecyltrimethoxysilane (OTS) modification of polymer substrates

The polymer substrate were initially cleaned by using acetone and dichloromethane under sonication for 10 mins and then dried. The cleaned film was immersed in 1 V/V% of OTS in dry dichloromethane(DCM) solvent for 15 minutes for surface coating of OTS. The physiadsorbed (silanized) substrate was rinsed with DCM and then dried. Then the film were cut into a small slides of pre-defined geometric area (1.5 cm × 2 cm) and used for cells made by sandwiching two physiadsorbed cellulose acetate polymer substrates. The LC cell and the silane coated film were dried at 100 °C for at least 1 h, thereafter cooled to room temperature. The silane modified film and the LC cell were used for LC alignment studies.

Chemical structure of the synthesized compounds was confirmed by a combination of Fourier transform infrared (FTIR) and nuclear magnetic resonance (NMR) spectroscopy. FTIR spectra were recorded using a FT/IR-4600 typeA spectrophotometer using KBr pellets for solid samples and ATR mode (ATR PRO ONE) for polymeric films. ^1^H NMR of the synthesized compounds was recorded using a Bruker Avance 400 MHz spectrometer (Bruker, Switzerland). For the NMR recording, samples were diluted using deuterated chloroform (CDCl_3_) and tetramethylsilane (TMS) was used as an internal standard. The LC properties of the synthesized compounds were investigated using polarizing optical microscopy (POM), differential scanning calorimetry (DSC) and X-ray diffraction studies (XRD). The textural observations were carried out by employing Olympus BX50 POM (Olympus Co., Japan) equipped with a Linkam LTS 420E (Linkam, UK) heating stage with T95-HS Link controller. Phase transition temperatures and associated enthalpy values were obtained from thermograms recorded on a Mettler Toledo DSC 822°, with a heating and cooling rate of 10 °C/s (accuracy ± 0.2 °C). XRD studies on powder samples were carried out using PANalytical, Empyrean diffractometer using Cu-K_α_ (λ = 1.54 Å) radiation. The samples were held in Lindemann capillaries with a diameter of 1 mm (Hampton Research, Aliso Viejo, CA, USA). The diffraction patterns of the sample were collected on a two-dimensional image plate (Marresearch, GmbH, Germany).

X-ray photoelectron spectroscopy (XPS) data were collected using a Thermo Scientific MULTILAB 2000 base system with a twin anode of Mg/Al (300/400 W) X-ray source with the electron gun spot size of <50 μm. The obtained spectral lines were corrected using C 1s spectral line appeared at a binding energy value of 284.6 eV by curve fitting method and the background fittings were also performed. Wettability of the unmodified, pre-treated and chemically modified cellulose acetate substrate was studied through CA measurement using water static sessile drop method. CA equipment (AST products Inc., USA) having an automated model of VCA Optima XE was employed for the measurements. Water CA was measured by dispensing a 10 µL drop of water on the respective surface and imaging the droplet immediately within 10 seconds of contact with the surface. The CA values were determined from the recorded images using the automated software of VCA Optima XE. In a typical experiment, CA values were measured at four different locations on a substrate and average of the four was considered for interpreting the nature of the surface (hydrophobic or hydrophilic) thereby to confirm the chemical modification on the surface.

Surface morphology of the as-received, pre-treated and LC anchored films were studied by AFM imaging and these experiments were performed by employing PicoSPM-Picoscan 2100 (Molecular Imaging, USA) instrument using NSC16 ultrasharp silicon cantilever tip (tapping mode).

### Data availability

All data generated or analysed during this study are included in this published article (and its Supplementary Information files).

## Electronic supplementary material

Supplementary Information

Vertically aligned nematic phase of 5CB over LC functionalized flexible polymer substrate
