# Supplementary material for: Vertical Alignment of Liquid Crystals Over a Functionalized Flexible Substrate
Source: Sci Rep. 2018 Jun 11;8:8891. doi: 10.1038/s41598-018-27039-3 (PMC5995910; doi:10.1038/s41598-018-27039-3)
Supplement: Supplementary file 1 — Supplementary Information [file 41598_2018_27039_MOESM1_ESM.docx]

Supplementary Information for

Vertical Alignment of Liquid Crystals Over a Functionalized Flexible Substrate

**B.Sivaranjini^a^, R. Mangaiyarkarasi^a^, V. Ganesh^b^ and S. Umadevi^*a^**

Department of Industrial Chemistry, Alagappa University, Karaikudi-630003, Tamilnadu, India

Electrodics and Electrocatalysis (EEC) Division, CSIR-Central Electrochemical Research Institute (CSIR-CECRI), Karaikudi-630003, Tamilnadu, India

e-mail: [umadevilc@gmail.com](mailto:umadevilc@gmail.com)

**Table of Contents**

| 1. Synthesis and characterization: scheme, experimental procedure and characterization data | Pages S2-S5 |
| --- | --- |
| 1. Mesophase characterization-DSC and POM | Pages S6-S8 |
| 1. XPS spectra of bare and modified substrate | Page S9 |
| 1. LC Alignment studies on LC modified cellulose acetate containing substrates –POM images | Page S10-S11 |

**Synthesis**

*Synthesis of 4-((4-decyloxy) benzoyloxy) benzoic acid,* ***A***

Thermotropic LC compound **A** was prepared following synthetic pathway shown in Scheme S1.

According to the scheme alkoxy benzoic acid was condensed with benzyl 4-hydroxybenzoate

to yield a two-ring ester. In next step, benzyl group was cleaved using catalytic hydrogenolysis, resulting in the required acid. Yield: 80%. Analysis: FT-IR(KBr) ʋ_max_ (cm^-1^): 3446, 3325, 3073, 2920, 2853, 1735, 1690, 1602, 1509, 1425, 1259, 1206, 1163, 1067, 1012, 843, 761, 661.^1^H NMR(400MHz, CDCl_3_) δ(ppm): 8.10 (d, 2H, Ar-H), 7.93 (d, 2H, Ar-H), 7.29 (d, 2H, Ar-H), 6.91 (d, 2H, Ar-H), 3.98 (t, 2H, Ar-O-C**H_2_**), 1.75 (quin, 2H, Ar-O-CH_2_-C**H_2_**), 1.30 (m, 14H, CH3-C**H2**-C**H_2_**-), 0.82 (t, 3H, -CH_2_-C**H_3_**).

**Figure S1.** Synthetic route followed to prepare the LC compound **A**


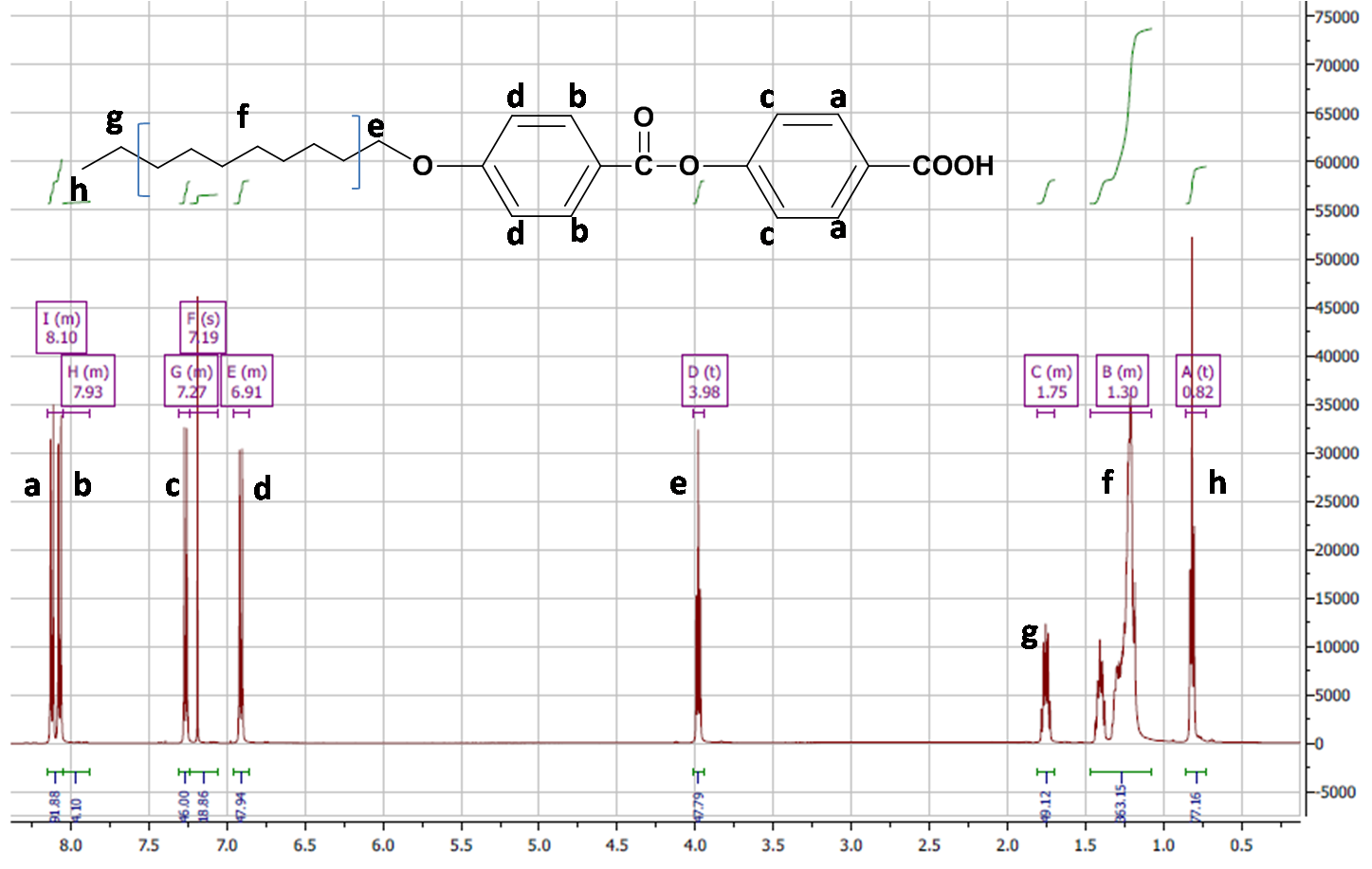


**Figure S2**. ^1^H-NMR of the compound **A**.


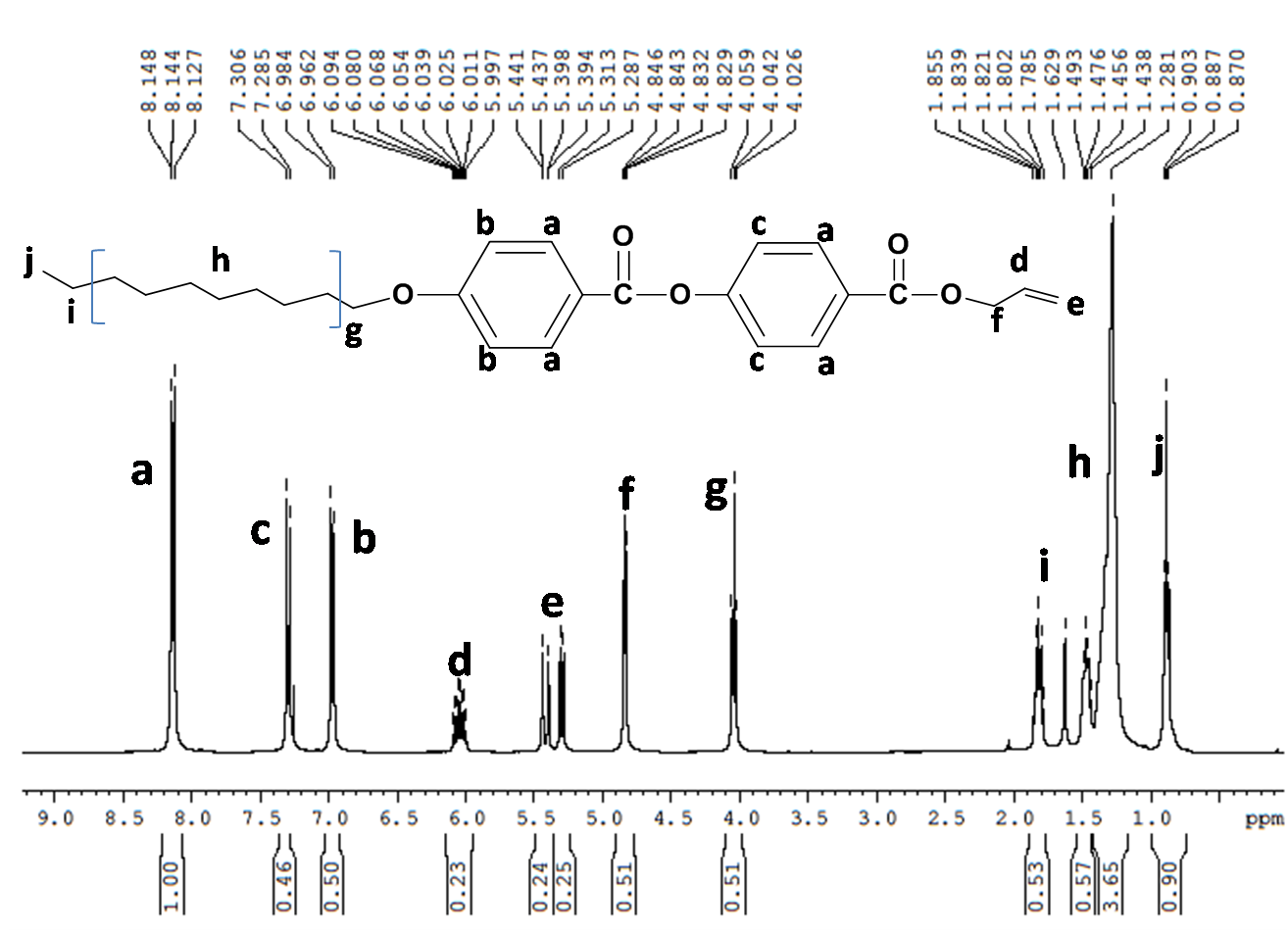


**Figure S3.** ^1^H-NMR of compound **B**


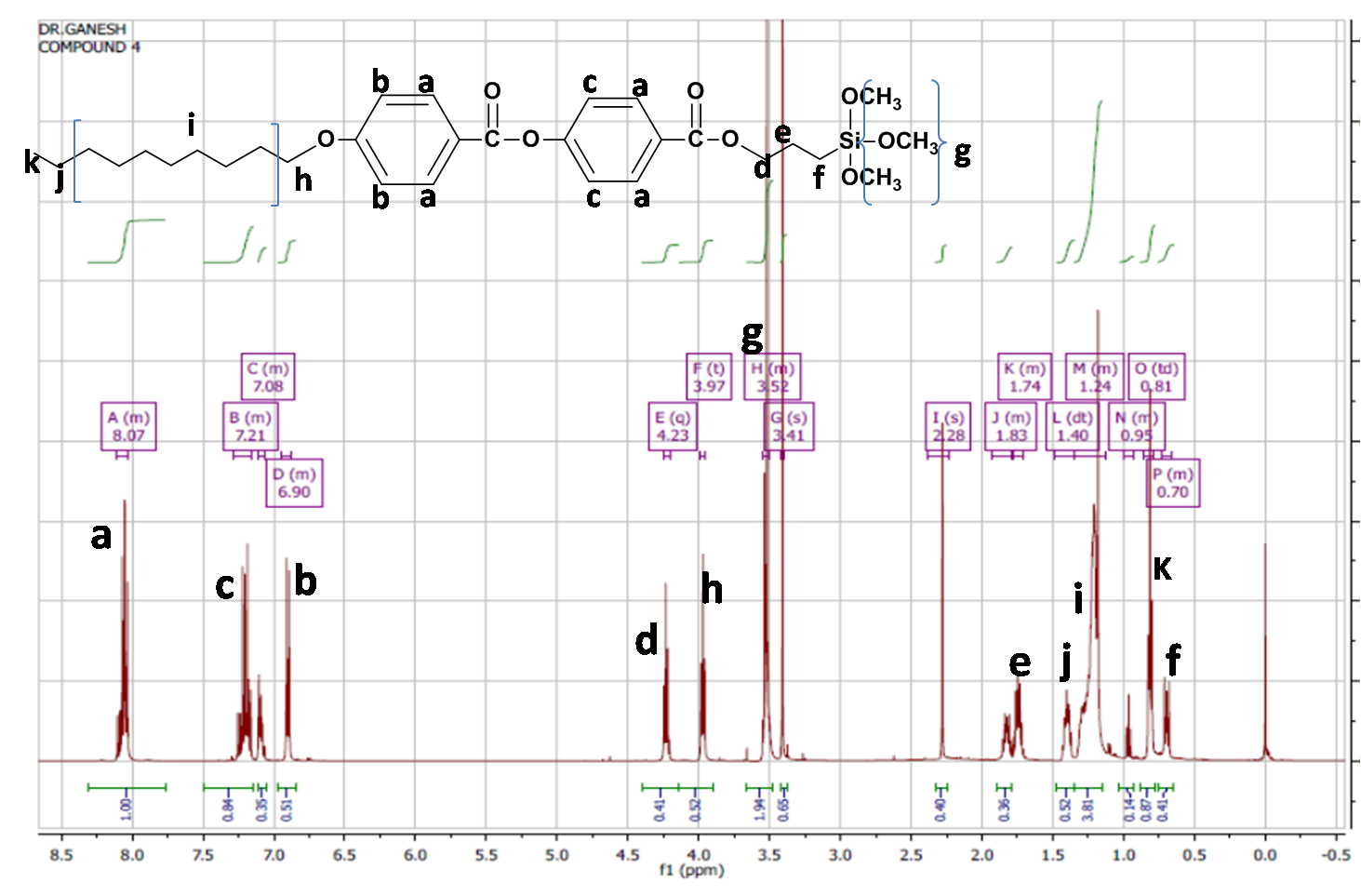


**Figure S4.**  ^1^H-NMR of compound **C**





**Figure S5.** A DSC thermogram obtained for compound **A.**





**Figure S6**. A DSC thermogram obtained for compound **B**.

**Table S1.**Transition temperature (°C) obtained for compounds **A** and **B**.

| S.No | Compound | Condition | Transition Temperatures |
| --- | --- | --- | --- |
| 1. | **A** | Heating | Cr 132°C SmC 198.5 N 224 I |
|  |  | Cooling | I 220°C N 178.7°C SmC 93.5°C Cr |
| 2. | **B** | Heating | Cr 57°C SmA 73.3°C I |
|  |  | Cooling | I 70.3°C SmA 31.0°C Cr |

Cr- crystalline phase, SmC- smectic C phase, SmA- smectic A phase, N- nematic phase, I- isotropic phase


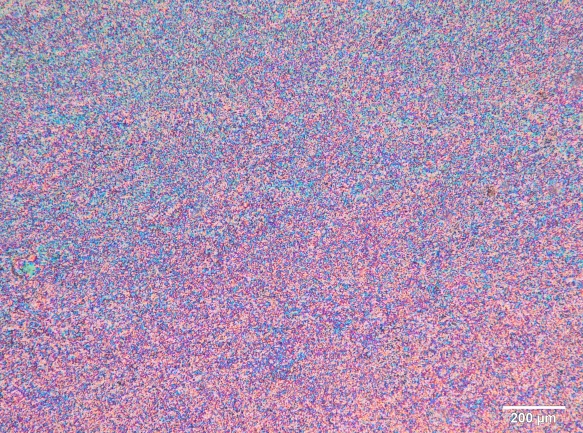

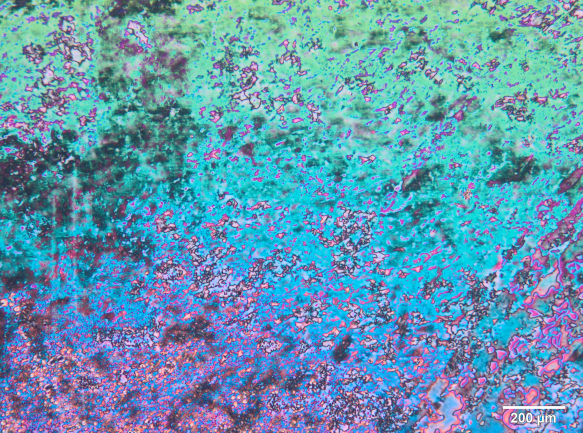


**b**

**a**

**Figure S7**. POM images displaying a grainy texture observed for smectic C phase of compound

**A** at 140°C (a) and marble texture obtained for nematic phase at 210°C (b), under

crossed polarisers.

After the hydrosilylation reaction, compound ‘**C’** was not isolated (attempts to purify the silane either through chromatography or by crystallization were unsuccessful) but used directly from the reaction mixture for the surface modification of substrate. However, the presence of **C** in the residue (obtained after evaporating the solvent from the reaction mixture) was confirmed through IR and NMR studies. Compound **C** also displayed the textural features of SmA phase (Fig. S8), however, it was difficult to identify the exact transition temperatures due to a broad melting and isotropic temperatures.


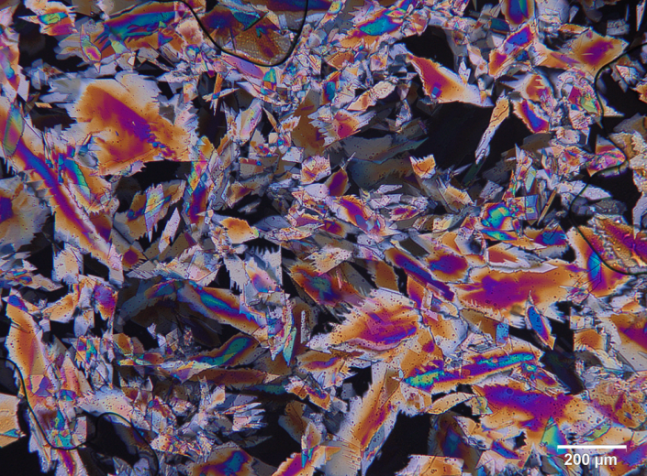


**Figure S8**. LC silane compound **C** in between a normal glass slide and a cover slip at 60°C.

**Figure S9**. Images of untreated (a) and LC modified (b)cellulose acetate containing polymer films

**XPS Spectra**

**
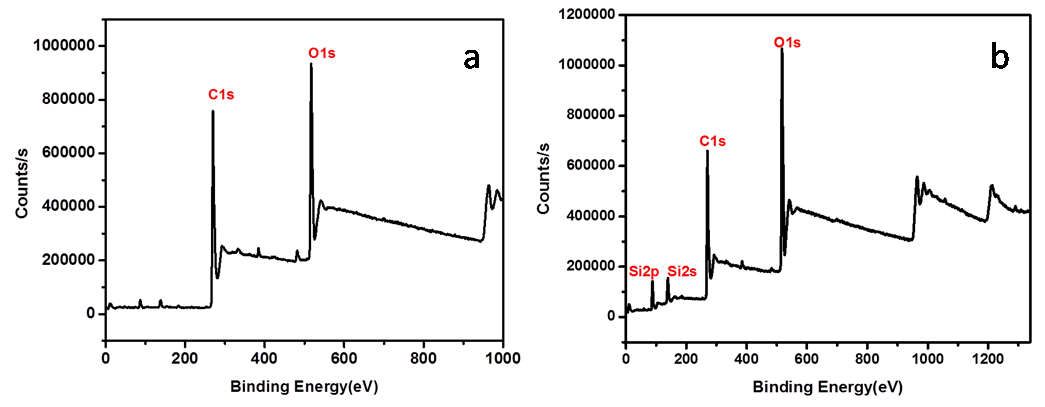
**

**Figure S10.** XPS elemental survey scan of (a) unmodified substrate and (b) LC modified substrate

**LC Alignment Studies on LC modified cellulose acetate containing flexible polymer substrate**


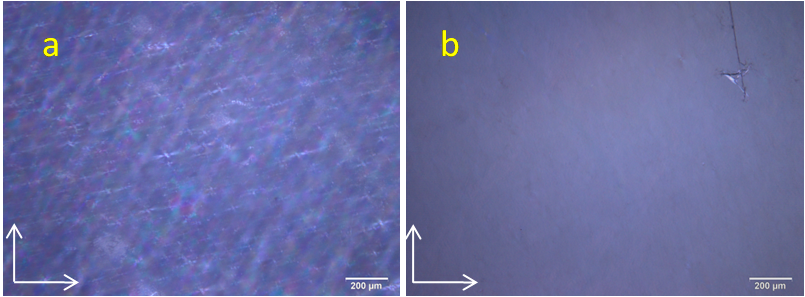


**Figure S11**. POM images of 5CB in a LC cell made up of two bare polymer films (a) and two LC

LC modified polymer films (b) separated by a 6μm spacer.

**Note**: LC modified cellulose acetate substrates were thermally stable at 80°C (investigated temperature) and were found to be chemically resistant upon treatment with common organic solvent namely dichloromethane, toluene, acetonitrile, butanone, acetone and ethanol (the films were dipped in the corresponding solvent for an overnight. After removing from the solvent, the films were found to be intact, no change in the appearance or softening of the film was observed. Chemical nature of the film was found to be unaltered which was confirmed through IR).

In order to assess the alignment of SmA phase of compound **B**, the polymer films containing the LC samples were heated using a hot stage and textures were observed under a POM. Prior to these experiments, polymer sheets were heated to 80°C for 18hrs and were found be intact after heat treatment. No change in the physical appearance was observed (such as softening of the film) and chemical nature was also found be unaltered as observed from IR. However, we could clearly noticed a change in the appearance of the substrate under POM upon heating which is probably due to alteration in the transparency of the film upon heating. Features of the polymer film (lines) are more expressed (dominant) in these images.
